# A motor neuron strategy to save time and energy in neurodegeneration: adaptive protein stoichiometry

**DOI:** 10.1111/jnc.14542

**Published:** 2018-09-21

**Authors:** Elisabetta Zucchi, Ching‐Hua Lu, Yunju Cho, Rakwoo Chang, Rocco Adiutori, Irene Zubiri, Mauro Ceroni, Cristina Cereda, Orietta Pansarasa, Linda Greensmith, Andrea Malaspina, Axel Petzold

**Affiliations:** ^1^ Centre for Neuroscience and Trauma Blizard Institute, Barts and the London School of Medicine and Dentistry Queen Mary University of London London UK; ^2^ Center of Genomic and post‐Genomic IRCCS Mondino Foundation Pavia Italy; ^3^ Department of Neurology China Medical University Hospital Taichung City Taiwan; ^4^ Department of Chemistry Kwangwoon University Seoul Korea; ^5^ Department of Brain and Behavioural Sciences University of Pavia Pavia Italy; ^6^ General Neurology Unit IRCCS Mondino Foundation Pavia Italy; ^7^ Sobell Department of Motor Neuroscience and Movement Disorders MRC Centre for Neuromuscular Diseases UCL Institute of Neurology University College London London UK; ^8^ Department of Neuromuscular Diseases MRC Centre for Neuromuscular Diseases Queen Square London UK; ^9^ The National Hospital for Neurology and Neurosurgery Queen Square London UK; ^10^ Moorfields Eye Hospital London UK; ^11^ Amsterdam UMC Departments of Neurology and Ophthalmology De Boelelaan Amsterdam NL

**Keywords:** ALS, energy, neurodegeneration, neurofilaments, stoichiometry

## Abstract

Neurofilament proteins (Nf) are a biomarker of disease progression in amyotrophic lateral sclerosis (ALS). This study investigated whether there are major differences in expression from *in vivo* measurements of neurofilament isoforms, from the light chain, NfL (68 kDa), compared with larger proteins, the medium chain (NfM, 150 kDa) and the heavy (NfH, 200‐210 kDa) chains in ALS patients and healthy controls. New immunological methods were combined with Nf subunit stoichiometry calculations and Monte Carlo simulations of a coarse‐grained Nf brush model. Based on a physiological Nf subunit stoichiometry of 7 : 3 : 2 (NfL:NfM:NfH), we found an ‘adaptive’ Nf subunit stoichiometry of 24 : 2.4 : 1.6 in ALS. Adaptive Nf stoichiometry preserved NfL gyration radius in the Nf brush model. The energy and time requirements for Nf translation were 56 ± 27k ATP (5.6 h) in control subjects compared to 123 ± 102k (12.3 h) in ALS with ‘adaptive’ (24:2.4:1.6) Nf stoichiometry (not significant) and increased significantly to 355 ± 330k (35.5 h) with ‘luxury’ (7:3:2) Nf subunit stoichiometry (*p* < 0.0001 for each comparison). Longitudinal disease progression‐related energy consumption was highest with a ‘luxury’ (7:3:2) Nf stoichiometry. Therefore, an energy and time‐saving option for motor neurons is to shift protein expression from larger to smaller (cheaper) subunits, at little or no costs on a protein structural level, to compensate for increased energy demands.

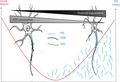

**Abbreviations used**
ALS: amyotrophic lateral sclerosis
ALSFRS‐r: amyotrophic lateral sclerosis functional rating scale ‐ revised
GLM: general linear models
MND: motor neuron disease
NfH: neurofilament heavy chain
NfL: neurofilament light chain
NfM: neurofilament medium chain
Nf: neurofilament proteins
PRB: progression rate at baseline
PRL: progression rate at last visit

Neurodegeneration is an irreversible process. Because neurons do not divide, disintegration leads to disappearance. Therefore, the defence against degeneration is essential. Multiple mechanisms of neuroprotection have been described (Nguyen *et al*. 2002; Xiong *et al*. 2004; Blasco *et al*. 2016). The common end pathway of failure of neuroprotection combines neuronal Ca^2+^ influx, oxidative stress, mitochondrial dysfunction and energy deficit. The possibility of endogenous neuroprotection by energy‐saving changes in specific structural protein expression has, to the best of our knowledge, not yet been considered.

We investigated this hypothesis in amyotrophic lateral sclerosis (ALS). Importantly, ALS is a neurodegenerative disorder with abnormal neuronal protein expression (Julien 2001). Clinically, the disease is highly heterogeneous but inevitably rapidly progressive and fatal (Turner and Swash 2015; Hardiman *et al*. 2017). Disease progression has been linked to axonal neurofilament proteins (Nf) stoichiometry in experimental models (Julien 1997). Neuro‐axonal degeneration causes release of Nf proteins to the adjacent body fluid compartment. Consequently, cerebrospinal fluid and blood Nf concentrations were found to be of diagnostic and prognostic value in ALS (Boylan, *et al*., 2013; Brettschneider *et al*. 2006; Lu *et al*. 2015b; De Schaepdryver *et al*. 2017; Gaiani *et al*. 2017; Gendron *et al*. 2017; Poesen *et al*. 2017; Steinacker *et al*. 2017). Over the past decade, Nf proteins have been validated as the most reliable body fluid biomarkers for neuro‐axonal degeneration in a range of diseases and experimental models (Lu *et al*. 2012; Comabella and Montalban 2014; Friese *et al*. 2014; Bacioglu *et al*. 2016).

The protein features of Nf isoforms are unusual in several aspects. The Nf proteins are intrinsically unstructured, have large polyanion tails, multiple phosphorylation sites, are able to self‐assemble into polymers and form intra‐ and extracellular aggregates in pathology (Dunker *et al*. 2001; Lu *et al*. 2011; Jucker and Walker 2013). The Nf heteropolymer consists of a light chain (NfL), a medium chain (NfM) and a heavy chain (NfH), (Petzold 2005; Yuan and Nixon 2016). The calculated theoretical mass of the Nf isoforms is lower than the real mass *in vivo* because of post‐translational modifications such as phosphorylation (Petzold 2005). In case of the central nervous system proteins, the Nf triplet is joined by α‐internexin. The phosphorylation of Lys‐Ser‐Pro (KSP) repeat motifs in Nf tails enriched in NfH and NfM proteins and their side‐arm protrusions mediate the interaction between neighbouring filaments and maintain axonal diameter through charge repulsion (de Waegh *et al*. 1992; Stevenson *et al*. 2011). The ratio in which the subunits polymerize to the neurofilament heteropolymer is referred as subunit stoichiometry. The Nf stoichiometry varies between species and adapts during development to cellular needs (Perrot *et al*. 2008). In humans, the Nf stoichiometric of NfL:NfM:NfH is 7 : 3 : 2 (Janmey *et al*. 2003). There is experimental evidence that altered Nf subunits isoform stoichiometry leads to impaired axonal transport and neurodegeneration (Collard *et al*. 1995; Julien 1997). Paradoxically, there were exceptions to this rule as demonstrated by Couillard‐Després *et al*. (1998), while new evidence suggests that modification of Nf stoichiometry may permit for stable Nf polymer formation and network composition over a large stoichiometric range (Beck *et al*. 2010). Rather than stoichiometry, the cause for disruption of the Nf liquid crystal gel network was found to be compartmental Nf accumulation and aggregate formation, another hallmark of ALS (Beck *et al*. 2012). There is also a need for the motor‐neuron to adapt protein transcription and translation with related energy demands within a limited time‐frame (Irvin *et al*. 2015), a critical process which may condition motor cell survival in neurodegeneration.

A limitation of previous work on human Nf isoform body fluid concentrations in ALS was that none of the studies investigated all three Nf subunits as elegantly summarized in a recent meta‐analysis (Li *et al*. 2016). Specifically, NfM was never quantified. Knowledge of all Nf isoforms is, however, a pre‐requisite for stoichiometric calculations. Consequently, previous work did not permit testing hypotheses on the potential relevance of altered human *in vivo* Nf subunit stoichiometry as suggested experimentally (Julien 1997, 2001).

In order to investigate the relevance of human *in vivo* Nf subunit stoichiometry and energy/time requirements in ALS, this study measured for the first time, simultaneously, all three Nf subunits from a clinically well‐characterized cohort of patients with ALS. Calibrated Nf subunit stoichiometry calculations were followed by well‐established Monte Carlo simulations. This permitted investigation of the radius of gyration for Nf side‐arms, independently from phosphorylation and ionic strength and to calculate time and energy requirement needed for Nf subunit protein expression. Taken together the data demonstrate that adaptive Nf subunit stoichiometry in ALS provides an elegant cellular strategy to maintain structural integrity while saving energy and time.

## Methods

### Standard protocol approvals, registrations and patient consents

Ethics were obtained from the East London and the City Research Ethics Committee 1 (09/H0703/27). All participants provided written consent when motor function permitted. Severely disabled patients gave witnessed verbal permission for a carer to sign on their behalf.

The study was not pre‐registered on ClinicalTrials.gov or any other institutional registration system. A more detailed in‐depth description of the methods of this translational paper as provided here can be requested from the corresponding author.

### Participant recruitment and sampling

This study included 60 ALS patients and 29 age‐ and sex‐matched neurologically healthy controls.

#### Inclusion criteria

Patients were diagnosed according to established diagnostic criteria (Brooks *et al*. 2000). Healthy controls were recruited from friends and relatives of the patients.

#### Exclusion criteria

Patients with neurological co‐morbidities known to affect Nf concentrations were excluded (Petzold *et al*. 2006a, 2010; Gaiottino *et al*. 2013; Disanto *et al*. 2015).

#### Clinimetrics

Disability was scored using the Functional Rating Scale‐Revised (ALSFRS‐r) at baseline and follow‐up. Symptom onset was defined as first patient‐reported weakness or speech impairment. Progression rate was calculated at baseline (PRB) or last visit (PRL) as 48 minus the ALS Functional Rating Scale‐Revised (ALSFRS‐r) score, divided by the disease duration in month from symptoms onset. Worsening of PRB/PRL by < 0.5 per month, by 0.5–1.0 points or more than 1.0 point per month was defined as slowly progressing ALS (ALS‐Slow), intermediately progressing (ALS‐Intermediate) or fast progressing ALS (ALS‐Fast) respectively. Disease stage was established at sampling and defined, based on ALSFRS‐R score, as early (score: 40–48), intermediate (score: 25–39) and late (score 24 and below).

### Sample processing

Plasma samples were processed and aliquoted within 1 h from collection and frozen at −80°C, following standard consensus procedures (Teunissen *et al*. 2009; Otto *et al*. 2012).

### Blinding procedures

A study ID was used to pseudonymize all samples. The study ID was used as a means for blinding. The laboratory team was blinded to demographic and clinimetric data. Clinical staff was blinded to laboratory results on biomarkers. The Modelling team was blinded to demographic and clinimetric data.

### Analytical procedures

An in‐depth description of the analytical approaches for quantification of neurofilament isoforms is provided with the Supplementary Material. In brief, plasma samples were used to quantify the neurofilament light (NfL), medium (NfM) and heavy (NfH) chains using previously published and validated quantitative methods (Lu *et al*. 2011; Gaiottino *et al*. 2013).

### Stoichiometry from neurofilament plasma concentrations in human

To calculate each neurofilament (Nf) isoform stoichiometry, we first converted all plasma Nf levels into g/L. We then determined molarity by dividing each concentration level by the individual isoform mass: 68 kDa for NfL, 150 kDa for NfM, 200 kDa for NfH^SMI35^ and 210 kDa for NfH^SMI34^ (Petzold 2005). Finally, Avogadro's number 6.0221415*10^23^ was used to calculate the number of particles. This provided us with a dimensionless variable suitable for a number of calculations relevant in this study, including energy and time requirements.

### Molecular model and Monte Carlo simulations of Nf stoichiometric assemble

Computer simulations based on the established coarse‐grained model (Zhulina and Leermakers 2007a,b; Chang *et al*. 2009; Stevenson *et al*. 2011) were performed. Using the reported molecular model, we performed canonical ensemble Monte Carlo (MC) simulations to investigate the interactions between parallel Nf backbones in regulating axonal diameter (Kumar *et al*. 2002). We considered Nf cross sections as two‐dimensional disks, with hard‐core area fractions equal to the experimentally obtained cross‐sectional area occupied by the Nf backbones. The simulation box had a dimension of 50 nm in the z direction (parallel to the Nf backbone) and 200 nm in both the x and y directions. The first residues in all Nf side‐arms were initially placed on a surface of the backbone with the interval of 1.6 nm in the z direction. The orientation of the first residues was chosen using a random number generator (rand function in the gnu fortran compiler, http://gcc.gnu.org/onlinedocs/gcc-4.8.0/gfortran/RAND.html), which was designed to generate an angle between 0 and 360 degree from the *x*‐axis. The location of the first residues in all Nf side‐arms was fixed during the whole simulation. To fit this model, we had to fix 31 Nf proteins per 50 nm unit length. We employed our calculated Nf‐isoforms stoichiometries at fixed ionic strength of I = 150 mM for both dephosphorylated and fully phosphorylated states to simulate the *in vivo* implications of ALS pathology on neuronal architecture in an ALS population. We included in the analyses, phenotypic variables as covariates primarily to evaluate the effect of disease progression on Nf stoichiometry.

### Energy and time requirements

To calculate time and energy requirements the following assumptions have been made: energy requirements for the addition of a single amino acid to a growing peptide chain were estimated as averaging at 5 ATP (Piques *et al*. 2009). The NfL protein consists of 543 amino acids, NfM of 916 amino acids and NfH of 1020 amino acids (Petzold 2015). Therefore, the number of Nf isoform particles was multiplied with the respective amino acid number and a factor of 5 to be consistent with the assumption made by Piques *et al*. (2009).

The time required to fold a 100‐amino acid protein was averaged at about 25 s (Balchin *et al*. 2016). Therefore, likewise to the ATP calculations the number of amino acids was taken as base for the calculations on protein expression.

### Statistical analysis

All statistical analyses were performed in SAS (version 9.4). This was the first study on NfM levels in ALS and therefore no sample size calculations could be performed prior to the exploratory analyses. Normality was tested graphically and using Shapiro–Wilk statistics. Gaussian data were presented as mean ± SD. Non‐Gaussian data were presented as median [interquartile range (IQR)]. For two‐group comparisons data with normal distribution were compared using the *t* test and non‐Gaussian data with the Mann–Whitney U test. For more than two‐groups comparisons of non‐Gaussian data, the Kruskal–Wallis test and General Linear Models (GLM) were used. Testing with GLM for multiple group differences had to reveal an overall significance in order to qualify for further comparisons. The *F*‐value and degrees of freedom are shown. All two‐way tests were two‐tailed. A *p*‐value of < 0.05 was considered statistically significant for single comparisons. For multiple comparisons, the Bonferroni‐corrected *p*‐value was used and specifically mentioned in the text.

## Results

### Study cohort

The baseline characteristics of the patient cohort are summarized in Table 1. The cohort was clinically heterogeneous in terms of age at onset, mean diagnostic latency, site of disease onset and gender predominance. Of note, diagnostic latency almost invariably coincided with disease duration at baseline sampling, reflecting an early inclusion in the study following diagnosis. Most of the patients had a diagnosis of definite or probable ALS at the time of inclusion, according to El‐Escorial criteria. The ALSFRS‐r score at baseline sampling, which reflects disease stage, averaged at 34.25 (Table**** 1).

**Table 1:** Baseline characteristics of patient cohort

Clinimetrics	Patients	Controls
Age at baseline sampling (years)		
Mean±SEM	64.67 ± 1.48	60.22 ± 1.73
Gender		
F/M	27/33	19/10
Ethnicity		
(% non‐Caucasian)	5%	2%
Smoking		
Yes/ex‐smoker/no	13/13/34	14/16
Site of onset		
Bulbar/limb/both	15/44/1	–
Age of onset (years)		
Mean± SEM	62.43 ± 1.49	–
Diagnostic latency (months)		
Mean± SEM	17.11 ± 2.91	–
El‐Escorial at sampling		
Definite/probable/possible/lab‐supported probable	14/29/11/6	–
ALSFRS_R score at baseline sampling		
mean±SEM	34.25 ± 1.02	–
Progression rate at baseline		
Mean±SEM	0.83 ± 0.1	–
Disease duration at baseline sampling (months)		
Less than 1 year/2 years/3 years/more than 3 years	18/16/11/15	–

ALSFRS‐r: ALS Functional Rating Scale‐revised; Progression Rate at Baseline (PRB) calculated as (48 ‐ ALSFRS_R at baseline)/time in months between onset of disease and the first visit.

The individual neurofilament data points for patients with ALS and control subjects are shown as a scatter plot in the Figure [Link], [Link], [Link].

### Subgroup analyses for disease progression

To assess whether clinical variables used to stratify our ALS patients may introduce a bias and affect neurofilament molar ratio calculations, we tested if any statistically significant difference was detectable in ALS subsets at the extremes of the three progression rates (i.e. fast progressor and slow progressors). Table S2 shows that there is no significant difference between fast and slow progressors in terms of age at onset and sampling, smoking habits or cognitive involvement. This implies there is no potential covariate for influencing change in stoichiometry between these two subgroups of patients.

The categorized progression rate (fast, intermediate, slow) at time of Nf isoform sampling, baseline, was significantly related to NfM (*F*_2,56_ = 3.88, *p* = 0.03), but not NfH (*F*_2,56_ = 0.13, *p* = 0.87) or NfL (*F*_2,55_= 2.13, *p* = 0.13) concentrations (Table S3). The Bonferroni‐corrected *p*‐value for the three progression groups calculates to 0.016. According to the Bonferroni corrections the *post hoc* analyses revealed significantly higher NfM levels in fast progressors if compared with slow progressors (*p* = 0.0074).

### Neurofilament stoichiometry

**Figure 1:**
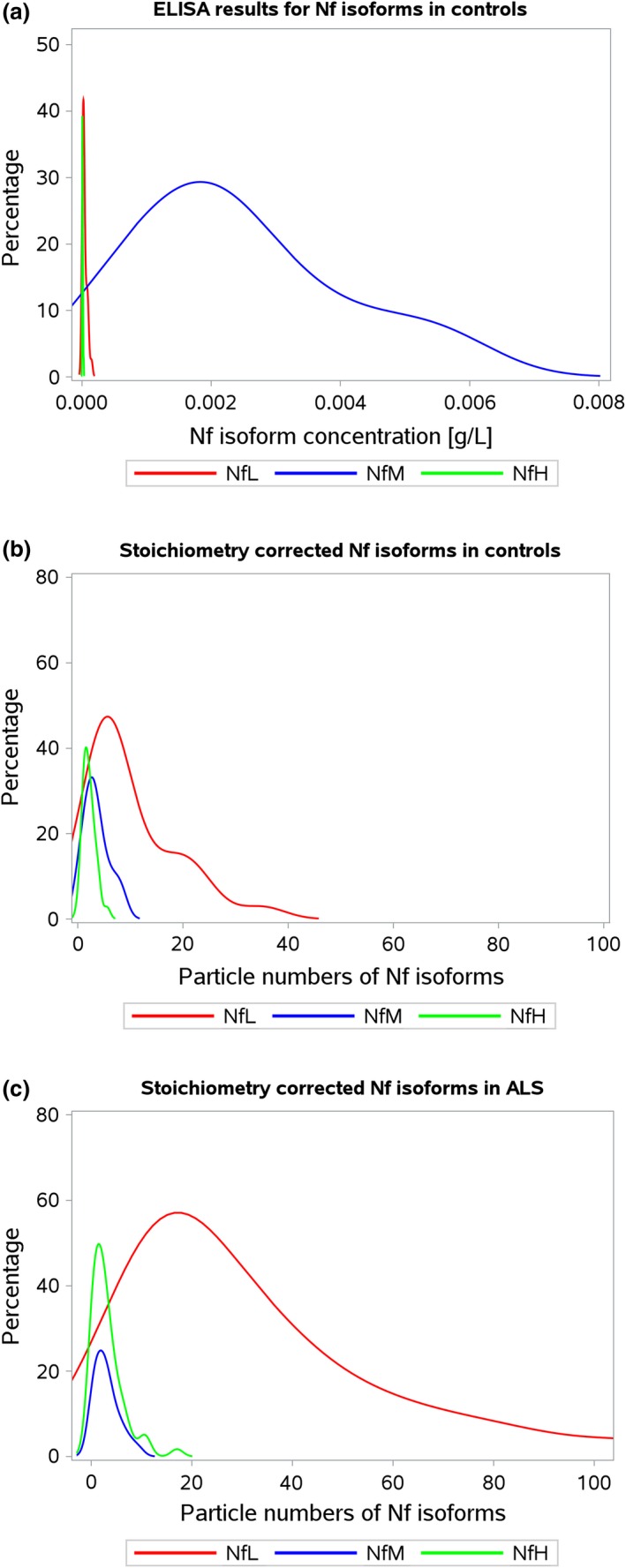
**Stoichiometry**. The *in vivo* neurofilament isoform plasma concentrations shown as kernel density plots of (a) the raw data of neurofilament isoform protein concentrations in control subjects (*n* = 29) reveal that a systematic error (an arbitrary Nf protein concentration rather than true molarity) is introduced by the ELISA method which skews the known stoichiometry of NfL:NfM:NfH of 7:3:2; (b) Neurofilament isoform protein concentrations in control subjects (*n* = 29) are shown after one‐point stoichiometric calibration of the absolute number of Nf isoform particles; (c) In patients with amyotrophic lateral sclerosis (ALS) (*n* = 60) the one‐point calibrated Nf isoform stoichiometry is right skewed. The ‘adaptive’ stoichiometry in ALS calculates to 24:2.4:1.6 for NfL:NfM:NfH. Colouring of the lines is red for NfL, blue for NfM and green for NfH.

The limitations of using individually, but not stoichiometrially calibrated neurofilament ELISA methods are shown in Fig. 1a. In healthy control subjects the absolute levels measured in pg/ml did not correspond to a biological stoichiometric relationship of the individual neurofilament isoforms in human (NfL:NfM:NfH of 7 : 3 : 2). Consequently, the use of different calibrators for the Nf isoforms introduced a systematic error to the dataset. In order to overcome this systematic error, the known stoichiometric relationship can be used for one‐point calibration if number of particles rather than absolute Nf isoform protein concentrations are used. Therefore, the average number of particles in control subjects were obtained from the Nf protein concentration as described in the method section. The dimensionless variables now permit for one‐point stoichiometric calibration. For each of the averaged Nf isoforms the correction factors were x = 7/(NfL particle count), y = 3/(NfM particle count), z = 2/(NfH particle count). Because of the non‐Gaussian distribution of the data, the resulting median particle count was used for the stoichiometric one‐point calibration. After calibration, the median NfL:NfM:NfH stoichiometric ratio calculated to 7:3:2 in controls (Fig. 1b) and 24 : 2.4 : 1.6 in ALS (Fig. 1c). The comparison of the stoichiometric frequency curves shows a proportional increase in NfL particles (*y*‐axis) and an overall increase in particle numbers (*x*‐axis). In contrast, the percentage contribution dropped for NfM and NfH (*y*‐axis) without overall less particle numbers (*x*‐axis). Taken together this suggests that in ALS the Nf heteropolymer is predominantly composed of the NfL isoform.

### Structure

**Figure 2:**
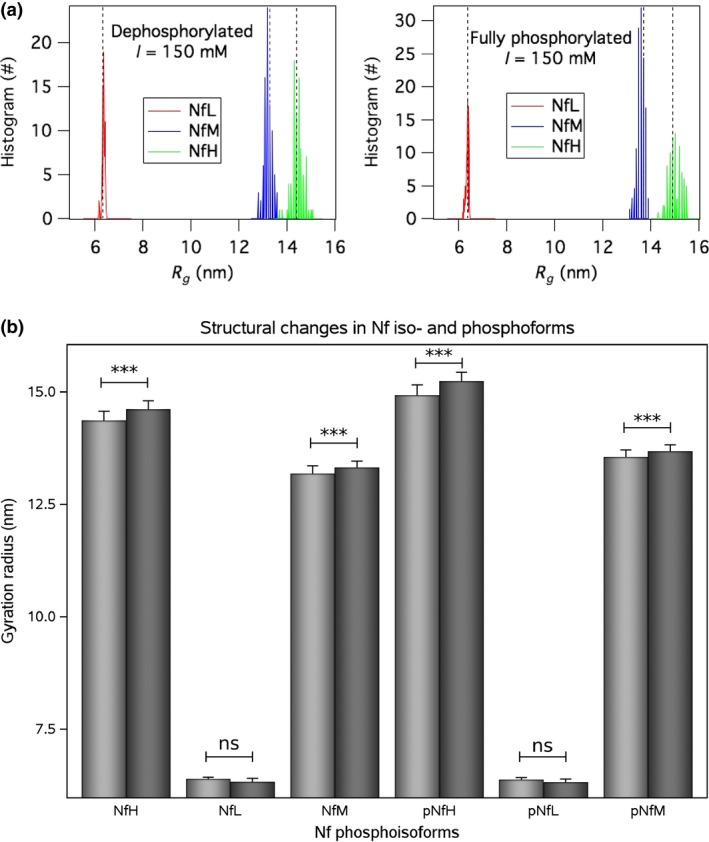
**Structure**. The Nf isoform gyration radius is shown at two phosphorylation states in controls (*n* = 29) and patients with amyotrophic lateral sclerosis (ALS) (*n* = 60). (a) Histogram of the Monte Carlo simulations of the gyration radius of Nf iso‐ and phosphoforms (*n* = 534) at a near physiological ion strength of I = 150 mM. The distribution is normal with a narrow spread resulting in a small standard deviation. The median is indicated by the dotted vertical reference lines. Colouring of the histogram bars is red for NfL, blue for NfM and green for NfH. (b) The averaged gyration radii were significantly smaller in ALS (light grey bars) if compared to controls (dark grey bars) for NfH and NfM, but not for NfL. The bar indicates the mean and the error bars show the standard deviation. Levels of significance are indicated as *p* < 0.0001 = *** and ‘ns’ = not significant.

Monte Carlo simulations did show that NfH is the main determinant for the radius of gyration in control subjects and patients with ALS (Fig. 2a, green histogram). Group differences in the gyration radius were minimal, but statistically significant (*F*_11,482_=25 496, *p *<* *0.0001, Fig. 2b). The *post hoc* analyses (*n* = 6, Bonferroni correct *p*‐value of 0.0083) showed a significant decrease in gyration in ALS compared with controls for non‐phosphorylated NfH (*p* < 0.0001) and NfM (*p* < 0.0001), as well as for their simulated phosphorylated states pNfH (*p*<0.0001), pNfM (*p* < 0.0001). No significant difference was, however, found for the gyration radius of either non‐phosphorylated NfL (*p* = 0.076) or phosphorylated NfL (*p* = 0.085) between ALS and controls.

### Energy

**Figure 3:**
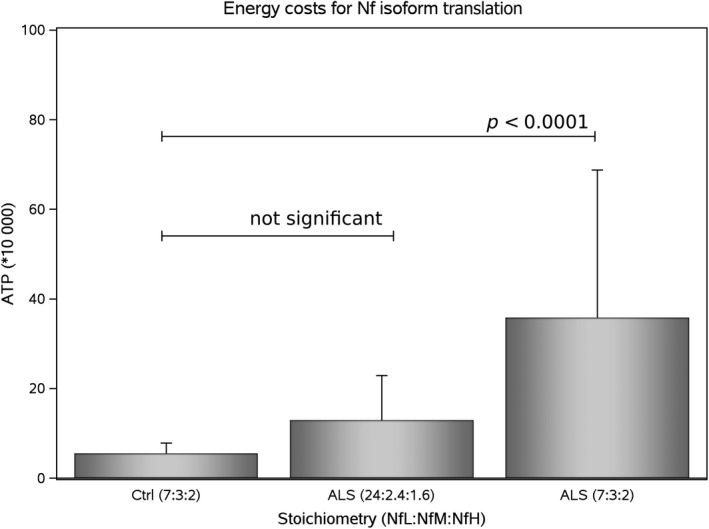
**Energy**. The calculated total ATP requirements for translation of the three Nf isoforms are shown for control subjects (*n* = 29) with a stoichiometry of NfL:NfM:NfH of 7 : 3 : 2. In patients with amyotrophic lateral sclerosis (ALS) (*n* = 60) the ‘adaptive’ Nf subunit stoichiometry (24 : 2.4 : 1.6) lead to a non‐significant increase in ATP (*p* = 0.197). By contrast, in ALS, a ‘luxury’ Nf subunit stoichiometry (7 : 3 : 2) would lead to significantly higher ATP requirements if compared to control subjects (*p* < 0.0001) or patients with ALS and ‘adaptive’ Nf subunit stoichiometry. The bar indicates the mean and the error bars show the standard deviation.

The ATP requirements for translation of all three Nf isoforms were significantly different between control subjects and ALS patients (*F*_2,140_ = 23.68, *p* < 0.0001, Fig. 3). The *post hoc* analyses (*n* = 3, Bonferroni correct *p*‐value of 0.0016) showed a significant increase in ATP requirements in patients with ALS if a stoichiometry of NfL:NfM:NfH of 7 : 3 : 2 was to be maintained compared with both control subjects and ALS patients with a Nf isoform stoichiometry of 24 : 2.4 : 1.6 (*p* < 0.0001 for both comparisons). There was however no statistical significant difference between control subjects and patients with ALS who had a stoichiometry of 24 : 2.4 : 1.6 (*p* = 0.197).

### Time

The shorter NfL isoform is more rapidly transcribed compared to the longer NfM and NfH isoforms. Total Nf isoform translation times in control subjects were 24 ± 11 h compared to 51 ± 40 h in patients with ALS and a Nf isoform stoichiometry of 24 : 2.4 : 1.6, which tripled to 148 ± 138 h if a Nf isoform stoichiometry of 7 : 3 : 2 was to be maintained. Again, the group difference was significantly different (*F*_2,140_ = 23.68, *p* < 0.0001) with a significant time penalty for maintaining a Nf isoform stoichiometry of 7:3:2 in ALS if compared with an adaptive stoichiometry of 24:2.4:1.6 in ALS (*p* < 0.0001) or control subjects (*p* < 0.0001).

### Clinical relevant findings for adaptive Nf stoichiometry on energy consumption

We hypothesized that the findings on the relationship between ATP requirements and Nf levels are of clinical relevance. Using less energy should be beneficial to patients. Higher energy demands in a progressive neurodegenerative condition should be detrimental. To test, this hypothesis we modelled the isoform stoichiometry in ALS. We compared the effect of the ‘adaptive’ Nf subunit stoichiometry of 24:2.4:1.6 with a ‘luxury’ stoichiometry of 7:3:2. These two scenarios were tested for their influence on comparing demographic and clinical variables.

#### Onset site

There was a significant difference in energy requirements for the two ALS Nf subunit stoichiometries comparing patients with bulbar versus limb onset sites (*F*_3,112_ = 10.98, *p* < 0.0001). With a ‘luxury’ Nf subunit stoichiometry the energy needs significantly increased in patients with bulbar onset (ATP 48 ± 51) if compared to patients with limb onset (ATP 31 ± 23, *p* = 0.01). In contrast, with an adaptive Nf stoichiometry, energy requirements were comparable between bulbar onset (15 ± 15) and limb onset (11 ± 7, *p* = 0.5).

#### Progression rate at baseline

There was a significant group difference for energy requirement according to the progression rate at baseline (*F*_5,110_ = 7.19, *p* < 0.0001). In patients with a ‘luxury’ Nf subunit stoichiometry, the energy needs were significantly increased with a fast progression rate (ATP 49 ± 32) if compared to a moderate (ATP 28 ± 24, *p* = 0.02) or slow progression rate (ATP 31 ± 38, *p* = 0.0078). In contrast, with an adaptive Nf stoichiometry, energy requirements were similar between fast (17 ± 10), moderate (9 ± 7) and slow (11 ± 12) progression (*p* > 0.05 for all comparisons).

#### Progression rate at follow‐up

At follow‐up energy requirements remained significantly high (*F*_5,110_ = 7.72, *p* < 0.0001) with a ‘luxury’ Nf subunit stoichiometry for a fast progression rate (ATP 49 ± 30) if compared with a medium (ATP 27 ± 22, *p* = 0.003) and slow progression rate (ATP 11 ± 13, *p* = 0.02). No such differences were observed with the adaptive Nf subunit stoichiometry (data not shown).

## Discussion

The main finding of this study was that the ‘adaptive’ *in vivo* Nf subunit stoichiometry observed in ALS had important advantages compared to a calculated ‘luxury’ Nf isoform stoichiometry. First, the calculated ATP requirements were significantly smaller with an adaptive stoichiometry of NfL:NfM:NfH of 24 : 2.4 : 1.6 compared with a ‘luxury’ Nf stoichiometry of 7 : 3 : 2. Second, it was computed that cellular expression of the adapted Nf heteropolymer was significantly faster (average 51 h) compared with the ‘luxury’ version (average 148 h). Third, the Nf heteropolymer structure was not compromised on a structural level.

The discussion of these data adds novel information to the literature on three levels.

First, present findings might explain why most of the literature shows a more substantial increase in body fluid NfL levels compared to NfH levels (Petzold 2005; Petzold *et al*. 2007; Li *et al*. 2016; Olsson *et al*. 2016; Zetterberg 2016). While consistent across a large disease spectrum, this observation has never been understood. One additional complicating factor has been that the full‐length NfL protein was found to be less stable compared to NfH (Schlaepfer *et al*. 1985; Wang *et al*. 1992; Koel‐Simmelink *et al*. 2011). Why should exactly the one Nf isoform which is most susceptible to proteolysis consistently be measured at high concentrations? The answer to this question comes from a very recent mass spectroscopy study by Brureau *et al*. (2017). The supplementary data of this study relate degradation of the full‐length NfL protein as shown by immunoblot, with appearance of a low‐molecular weight NfL peptide in mass spectrometry. Presence of such a peptide now also permits to reconcile analytical data on the absence of a hook effect for NfL with what can be observed for the full lengths NfH and NfM chains (Lu *et al*. 2011; Gaiottino *et al*. 2013; Kuhle *et al*. 2016). Likewise, there are well‐known proteolytic peptide fragments for NfH (Lu *et al*. 2011; Petzold *et al*. 2011). Still, presence of an analytical suitable NfL proteolytic breakdown product may not completely explain the disproportional increase in NfL compared with NfH in the study. Based on present data, it is suggested that the ‘adaptive’ Nf subunit stoichiometry, which over‐expresses NfL 15 times compared to NfH, is one important reason for higher body fluid NfL levels compared to NfH (Petzold 2005; Petzold *et al*. 2007; Li *et al*. 2016; Olsson *et al*. 2016; Zetterberg 2016). Future studies may consider quantification of Nf subunit mRNA levels.

Second, the raw data on Nf isoform concentrations calls attention to the fact that all of the immunoassays were calibrated to an arbitrary Nf isoform protein standard. Typically, the Nf proteins are purified from animal cadavers by, for example, HPLC (Karlsson *et al*. 1987). Most companies then use a total protein assay (e.g. Bradford) to quantify their product. Therefore, a ‘systematic error’ is introduced for quantification of each Nf isoform. By ‘systematic error’ we mean that for none of the available Nf isoform protein standards a calibration has been performed to the exact number of moles Nf per volume, known as molarity. In contrast, all Nf assays to date make use of what chemists had done in the past, to quote the Nf concentrations as (weight of solute)/(volume). Therefore, we do not really know about the number of moles of Nf, but we rest reassured that all tests are done to a protein standard calibrated to reasonable standards within what is technically feasible with current technology. This ‘systematic error’ does not affect single protein comparisons between groups as done for the large biomarker literature on neurofilament levels in disease. The ‘systematic error’ becomes substantial when considering stoichiometry, because the moles per solute remain unknown. In addition, there are obvious batch‐to‐batch variations, need for re‐calibration of the standard curve and a significant inter‐laboratory variability which have not been resolved (Petzold *et al*. 2010; Miller *et al*. 2016). To overcome the issue of variability, all samples in this study were batch‐analysed as recommended by Petzold *et al*. 2010;. Present data clearly demonstrate that in addition to the issues with individual Nf isoform immunoassays, the methods do not yet permit for a straightforward assessment of the *in vivo* Nf isoform stoichiometry, found to be so important in experimental models (Collard *et al*. 1995; Julien 1997, 2001; Couillard‐Després *et al*. 1998). Consequently, the amount of Nf isoforms measured cannot directly be compared for stoichiometric comparisons using the units provided. In this case, it is mathematically not correct to perform stoichiometric calculations. The solution to the situation was conversion into a dimensionless variable. A dimensionless variable permits for mathematically clean calculation of ratios. The dimensionless variable in the present context is the ‘particle number’. The computational approach presented here is suitable to implement a cross‐assay calibration based on stoichiometry.

Third, the literature on the diagnostic and prognostic value of Nf isoform levels is evolving (Brettschneider *et al*. 2006; Bowser *et al*. 2011; Ganesalingam *et al*. 2011; Boylan *et al*. 2013; Lu *et al*. 2015b; Steinacker *et al*. 2016, 2017; Weydt *et al*. 2016). Due to the cross‐sectional nature of many studies, more work had been done on the diagnostic value of Nf isoform levels in ALS with several papers being published while this manuscript was under review (De Schaepdryver *et al*. 2017; Feneberg *et al*. 2018; Rossi *et al*. 2018; Thompson *et al*. 2018; Verde *et al*. 2018). Correlative analyses of Nf isoform levels with clinimetrics such as disease progression are particularly severely affected. It seems plausible that the ‘adaptive’ Nf subunit stoichiometry changes with pathology and during different stages of neurodegeneration. Importantly, there is a large range of functional Nf subunit stoichiometries (Couillard‐Després *et al*. 1998; Julien 2001; Petzold *et al*. 2009; Kim *et al*. 2011; Beck *et al*. 2012). Present data revealed that the computed energy requirements for maintaining a Nf heteropolymer were more informative compared to individual Nf subunits. This does make biological sense. There is an emerging consensus of neurodegeneration been driven by depletion of ATP resources (Dutta *et al*. 2006; Mahad *et al*. 2009; Burté *et al*. 2015; Warne *et al*. 2016). Future studies will need to test this hypothesis by including biomarkers for mitochondrial function and ATP metabolites (Amorini *et al*. 2009; Lazzarino *et al*. 2010).

There are limitations to the study. Present modelling of time and energy requirements for Nf proteins production is limited by the fact that we can account only for 80% of energy expenditure, which is used for oxygen consumption coupled to ATP synthesis, while approximately 25–30% goes towards protein synthesis, 19–28% of which by the Na(+)‐K(+)‐ATPase (Rolfe and Brown 1997). Our calculation does not embrace protein folding and transfer, which is clearly going to contribute to the overall energy requirements and be more energy consuming for the larger NfH and NfM subunits compared with the leaner NfL. However, protein folding is a dynamic state and strongly influenced by charge properties and the external milieu. Phosphorylation and de‐phosphorylation of Nf can occur at low energy expenditure in the axon. Assembly of the Nf heteropolymer and post‐translational modifications in the neuron are likely to be more ATP demanding. Therefore, in addition, compartmentalization will need to be taken into account in future experimental studies on adaptive protein stoichiometry. The role of Nf stoichiometry and post‐translational modification in the modelling of axonal structure is constantly been revised by emerging experimental evidence. A recent gene replacement study revealed that the phosphorylation of mouse NfM KSP repeats does not affect axonal architecture, challenging the conventional viewpoint on the structural role of Nf phosphorylation (Stevenson, Chang, & Gebremichael, 2011). The interpretation of the stoichiometry data in ALS is also limited by the lack of a full understanding of the specific role of Nf in the regulation of inter‐filament distances and axonal diameter.

Next, the Monte Carlo simulation also shows how the radial extension of the core neurofilament structure is affected by the NfL shift and change in side‐arms, suggesting that altered molar concentrations have a structural effect and may also influence the M‐KSP phosphorylation and ultimately the axonal diameter and function. The simulations and immunoassays do not permit to analyse the potential for compartmentalized Nf protein aggregate formation, a hallmark of neurodegeneration in ALS (Julien 1997, 2001; Julien and Mushynski 1998). Furthermore, immunoassays are based on quantification of proteins by recognition of certain epitopes only. This limitation is, however, inherent to the entire biomarker literature. Body fluid biomarker levels are affected by stability, solubility among other factors and only ever will provide indirect information on true tissue protein levels. Likewise, it is presently impossible to tell a degenerating peripheral from a degenerating central neuron as source for elevated plasma NfL, NfM and NfH levels.

In addition, the balancing effort between energy and structure described here in a rapidly developing neurodegenerative process like ALS, may be a far more widespread adaptive mechanism and underpin any change in the brain which supports plasticity and recovery from injuries of different types. Clinical heterogeneity in ALS remains an important challenge (Turner and Swash 2015).

Finally, this study did not consider the presence of neurofilament auto‐antibodies which can cause epitope masking and thereby interfere with immunoassay protein quantification (Petzold 2005). There is evidence that the non‐rising trajectory of NfL in bio‐fluids during disease progression may simply reflect more efficient clearance by the immune response, as raising anti‐NfL antibodies in a more advanced disease stage has been reported in ALS (Puentes *et al*. 2014).

From a mechanistic viewpoint, further studies are needed to gain more insight into the role of Nf isoforms in neuronal injury using, for example, RNA antisense and genetic knock‐out. In addition, there is a clear need for high‐density longitudinal data on the likely variable nature of the density functions of the various Nf isoforms. With emerging SIMOA technology (Kuhle *et al*. 2016) this should become feasible on small volume plasma samples as typical for mouse models (Lu *et al*. 2012). On the other hand, to deepen our understanding of Nf as biomarkers of disease progression, it will be important to develop a Nf multiplexing approach where a snapshot of Nf isoforms concentration may be available in the same sample tube. This approach will also need to overcome the limitation of a non‐molarity driven antigen‐specific calibration. Also, future investigations should address whether changes in the *in vivo* Nf stoichiometry and effects on the Monte Carlo simulations structure of Nf side‐arms correlate not only with the disease phenotype but also relate to stage of disease progression in longitudinal studies.

In conclusion, this is the first study to simultaneously quantify in vivo all three protein isoforms of the obligate Nf heteropolymer, NfL, NfM and NfH. Detailed stoichiometric calculations and structural computations suggest that an ‘adaptive’ Nf subunit stoichiometry has important energetic advantages for the motor‐neuron and is also a time‐efficient strategy in this rapidly progressive neurodegenerative disorder.

## Author contribution

EZ, C‐HL, RA and AP performed the laboratory work; EZ, C‐HL and AM obtained the consent of patients and collected clinical data; RA, IZ, MC, CC, OP, AP, C‐HL, RA, AM, LG and AP provided intellectual content. CR and AP performed the statistical analyses and provided the figures. YC and RC performed Monte Carlo simulations and analysed the simulation data. AM, AP, EZ and C‐HL had the idea to the study. AM and AP had final equal contributions to the writing of the article. All authors revised the final version. All authors had access to the data.

## Supporting information


**Figure S1.** The concentrations of neurofilament isoforms are shown for patients with ALS and controls for (A) NfL [g/L], (B) NfM [g/L] and (C) NfH [g/L].

**Table S1.** Protocol for the NfH ELISA.
**Table S2.** Clinical features and exposure: fast versus slow progressors. Continuous variables were presented with median (IQR).
**Table S3.** Summary of plasma concentration of NfL, NfM, and NfH in ALS patients and in controls. Plasma Nf levels of controls and ALS clinical subgroups were presented as Median (IQR).
