# Supplementary figures and images for: A motor neuron strategy to save time and energy in neurodegeneration: adaptive protein stoichiometry
Source: J Neurochem. 2018 Sep 21;146(5):631–41. doi: 10.1111/jnc.14542 (PMC6175430; doi:10.1111/jnc.14542)

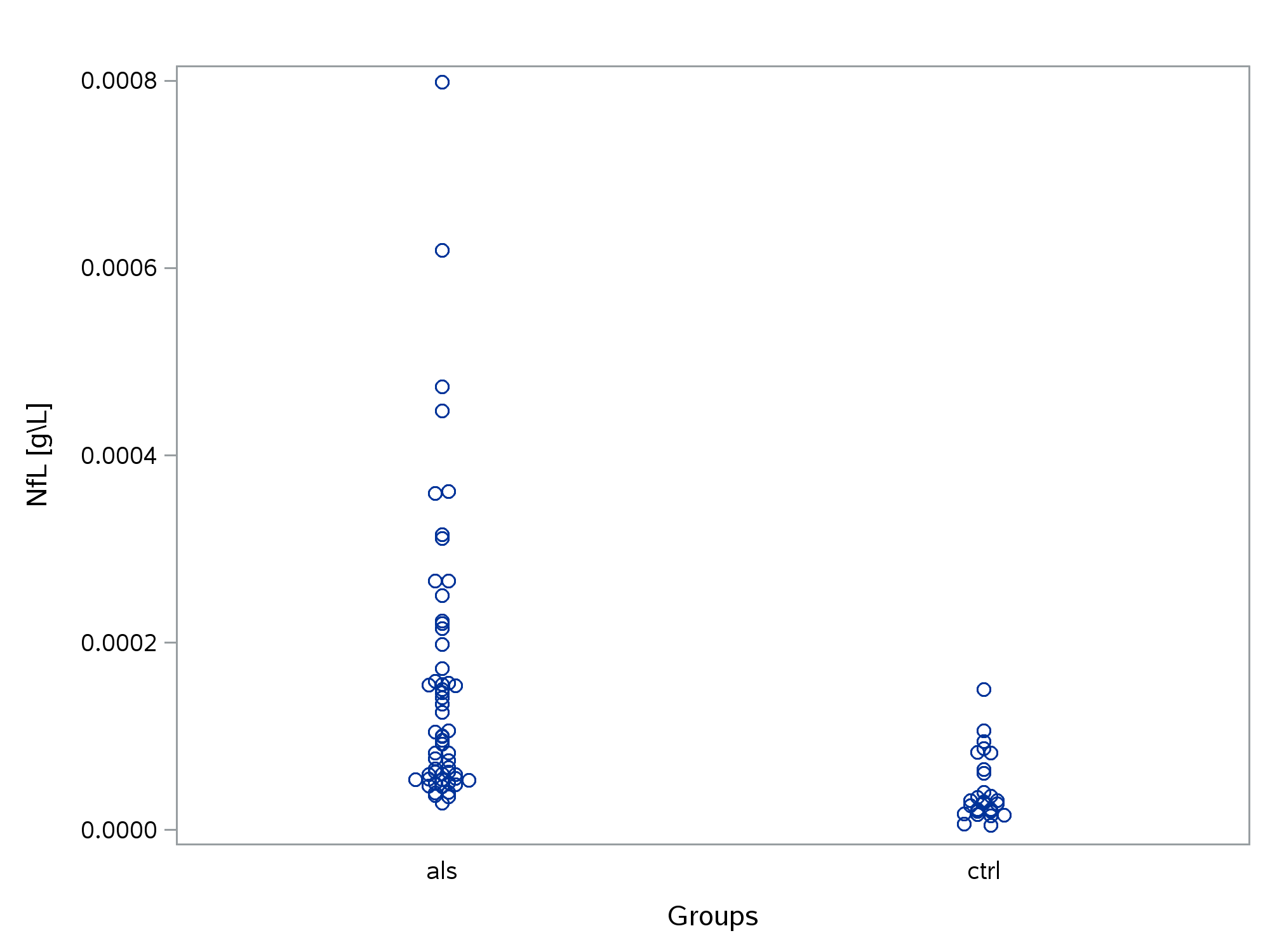

Supplement: Supplementary file 1 — Figure S1. The concentrations of neurofilament isoforms are shown for patients with ALS and controls for (A) NfL [g/L], (B) NfM [g/L] and (C) NfH [g/L]. [file JNC-146-631-s001.png]

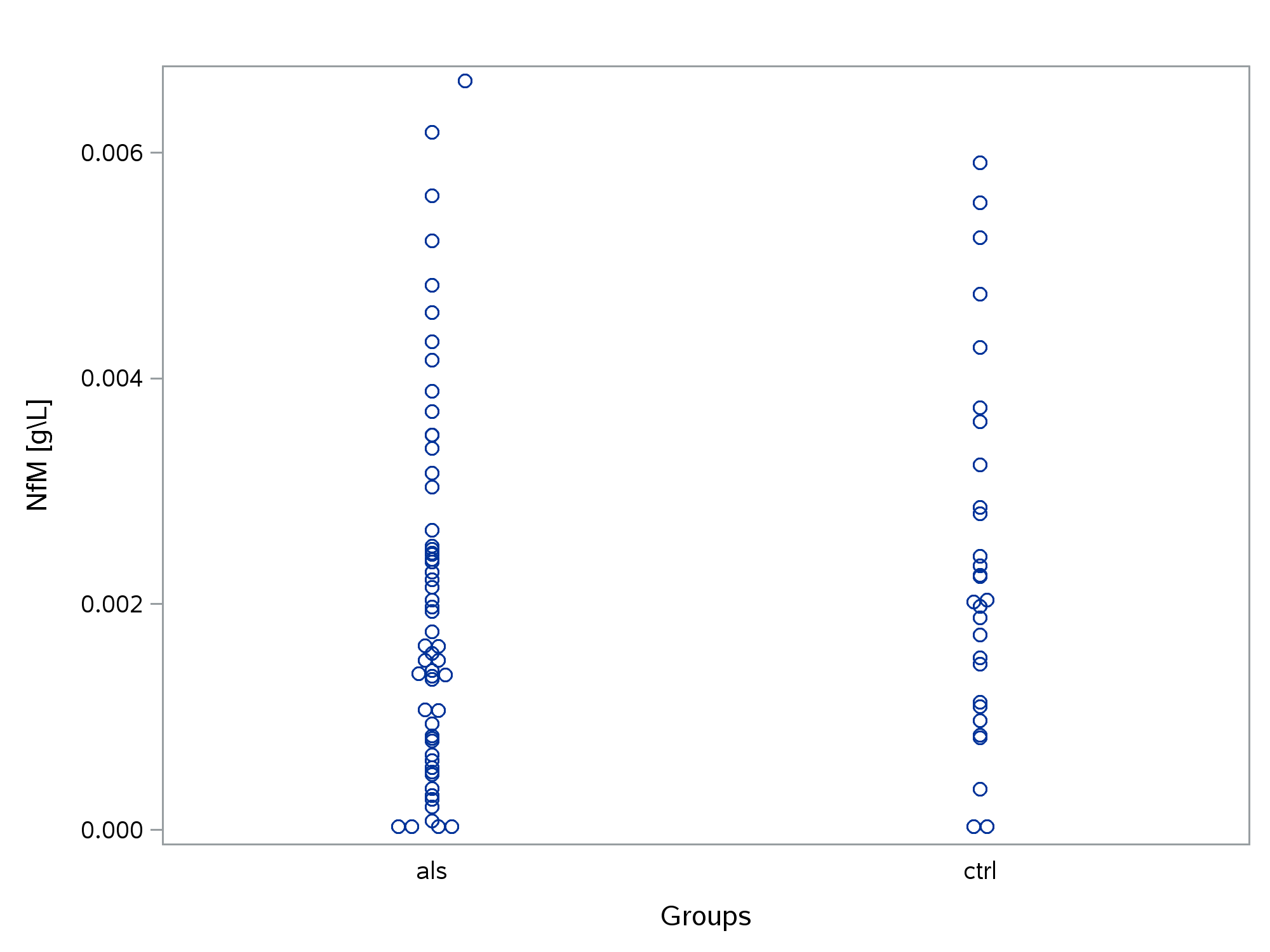

Supplement: Supplementary file 2 [file JNC-146-631-s002.png]

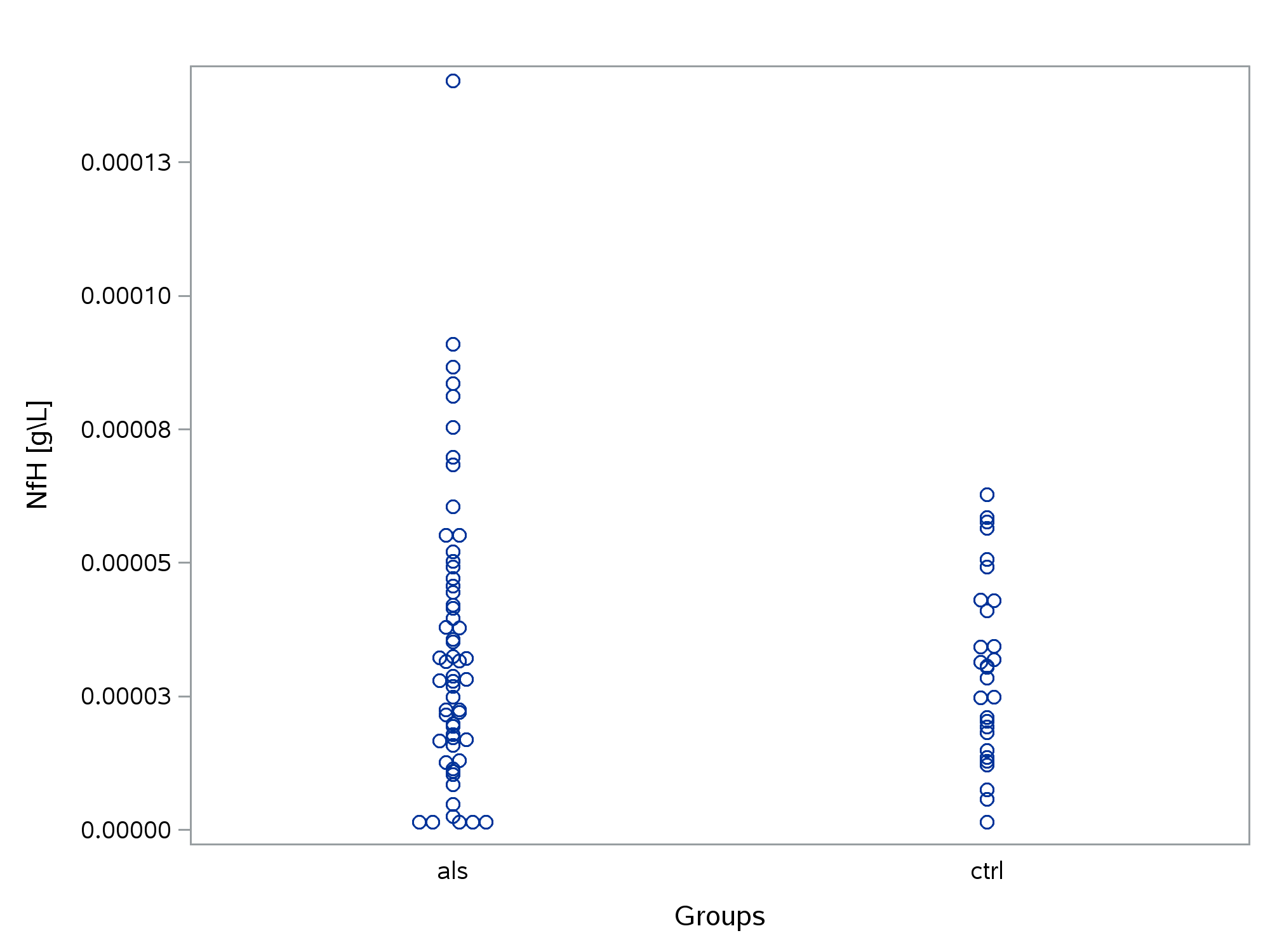

Supplement: Supplementary file 3 [file JNC-146-631-s003.png]
