# Supplementary material for: A motor neuron strategy to save time and energy in neurodegeneration: adaptive protein stoichiometry
Source: J Neurochem. 2018 Sep 21;146(5):631–41. doi: 10.1111/jnc.14542 (PMC6175430; doi:10.1111/jnc.14542)

Supplementary material to the manuscript:

A motor neuron strategy to save time and energy in neurodegeneration: adaptive protein stoichiometry

Elisabetta Zucchi, Ching-Hua Lu, Yunju Cho, Rakwoo Chang, Rocco Adiutori, Irene Zubiri, Mauro Ceroni, Cristina Cereda, Orietta Pansarasa, Linda Greensmith, Andrea Malaspina, Axel Petzold

Supplementary material on the analytical methods

Neurofilament quantification

Quantification of neurofilament isoforms was restricted to the light chain (NfL), the medium chain (NfM) and the heavy chain (NfH). Quantification of neurofilament phosphoforms was restricted to the phosphorylated heavy chain (NfH^{SMI35}) and hyperphosphorylated heavy chain (NfH^{SMI34}). All quantitative data were reported as pg/mL or ng/mL as detailed in the following three sections. For all tests all solvents were prepared with deionized ultrapure water to a 18 Mωm cm in resistivity at 25°C.

Other reagents used to assess the three of neurofilament isoforms were bovine serum albumin (BSA), ethylenediaminetetraacetic disodium salt (EDTA), NaCl, phosphate buffered saline, pH 7.5 (PBS), tris base and Tween 20 of analytical grade (Sigma-Aldrich, Saint Louis, MO). More detailed Research Resource Identifiers (RRIDs, scicrunch.org) were not yet available.

Neurofilament light chain (NfL)

The NfL analysis was performed as described in the methods paper by Gaiottino *et al.*, 2013. All antibodies and protein standards were purchased from UmanDiagnostic, Sweden and used within two weeks of delivery. The assay was performed by first coating 96-well plate including integrated screen-printed carbon ink electrodes on the bottom of the wells (96-well SECTOR standard plates, Meso Scale Discovery, Gaithersburg, USA with 30 µL of the mouse Capture monoclonal antibody (clone 47:3 from reference) diluted in carbonate bicarbonate buffer (pH 9.0, Sigma-Aldrich). The plates were put on a plate shaker at 800 rpm for 3 seconds. Then the plates were incubated overnight at 4C. The following day the plates were rinsed 3 times with 200 µl of wash buffer (0.1% Tween20 in tris buffered saline (TBS), pH 7.5) per well. Next 100 µl of blocking buffer (3% milk in TBS) were added per well. The plates were sealed and inclubated on a plate shaker (800 rpm) at room temperature (RT) for 1 hour. Whilst the plate was incubated protein standards were prepared fresh. Bovine lyophilized NfL (UmanDiagnostics, Sweden) were diluted in TBS containing 1% Bovine Serum Albumin, 0.1% Tween 20, pH 7.5 and ranged from 0-10,000 pg/ml. Next,

the plates were rinsed 3 times with 200 µl of wash buffer as before. Then 25 µl of Heteroblock (Omega Biologicals) were added to the standard and blank well at a concentration of 300 µg/ml. To the sample wells 25 µl of Heteroblock were added at a concentration of 600 µg/ml. To the prepared plate 25 µl of standard and sample were added in duplicates. The wells were sealed and incubated on the shaker for 2 hours at RT. Following this, plates were washed as before 3 times. Next, the detecting mouse biotinylated detector antibody (2:1, UmanDiagnostic, Sweden) was diluted in TBS containing 1% Bovine Serum Albumin, 0.1% Tween 20, pH 7.5. Of this 25 µl were added to each well. Plates were sealed and incubated on the plate shaker at RT for 1 hour. After a final washing cycle (3x), 25 µl of SULFO-TAG^{TM} labelled streptavidin (MSD, Gaithersburg, USA) was added to 150 µl of read buffer (MSD, Gaithersburg, USA) to generate electrochemiluminescence and the signals were detected with a SECTOR Imager 2400 camera (MSD, Gaithersburg, USA). The quality control sample was used to record the inter-assay variation and for additional one-point calibration as described [Petzold *et al.* 2010].

Neurofilament medium chain (NfM)

We have used a commercially available ELISA kit (Cloud Clone Corp., product no. SEB326Hu) to measure plasma NfM, based on a sandwich ELISA with two different polyclonal antibodies against NfM. Antibodies, protein standards as well as detection and dilution reagents were all provided by the kit; standard, detection reagent A and B and the 96-well strip plate have been kept stored at -20° C upon receipt till use within one month. Standard curve was reconstituted by diluting Standard (NfM provided by the kit at a concentration of 4000 pg/mL) to the highest standard fixed at a concentration of 1000 pg/mL, then performing a double dilution curve till the concentration of 31.2 pg/mL. No previous evaluation of pre-analytical variables in a complex matrix as plasma was available for NfM. We therefore assessed NfM levels after serial plasma dilutions with 0.01 mol/L phosphate buffered saline (PBS, as recommended by the manufacturer) and found a hook effect similar to NfH (Lu *et al.*, 2011) (*data not presented*). In order to overcome this effect, we tested different conditions as described elsewhere (Lu *et al.*, 2011) to achieve parallelism. For NfM we found that parallelism was achieved with a dilution factor of more than 1:8. Therefore, plasma samples were processed at 1:10 with 0.01 mol/L PBS. If not taken into account the hook effect can cause underestimation of the correct Nf concentration.

After having plated 100 µl of the standard, blank, and samples in duplicate in each well, the plate was incubated for one hour at 37°C on the shaker. Next, the liquid was removed and 100 µl detection reagent A, provided by the manufacturer, was added to each well, leaving the plate in incubation for one hour at 37°C. Liquid was consequently removed again, and 350 µl wash solution were added to each plate by a multichannel pipette, let it rest a couple of minutes, and then removed again; this step was performed thrice. After the last wash, 100 µl detection reagent B were added to each well and the plate was incubated for 30 minutes at 37°C. Next, the washing process was repeated 5 times as described above. After the last wash, 90 µl Substrate Solution were added to each well, and the plate was incubated for 15 minutes at 37°C with light shedding. Finally, 50 µl of Stop Solution were added to each well and, after gentle mixing, the plate was run on Microplate Reader at 450 nm. %.   The quality control sample was chosen from a plasma sample with high NfM concentration. The quality control sample was used to record the inter-assay variation and for additional one-point calibration as ALREADY described [Petzold *et al.* 2010]. Intra-assay coefficient of variation (CV) was always below 20%, with an average value at 6.91%, while inter-assay CV was found at 2.94%. The detection curve of the kit was fitted between 15.1 and 1,000 pg/mL, with an overall analytical sensitivity above 6.1 pg/mL.

Neurofilament heavy chain (NfH)

Regarding NfH, an in-house ELISA for hyper-phosphorylated NfH (NfH^{SMI34}), variably phosphorylated NfH (NfH^{SMI35}) and for the sum of the two were employed as reported in Lu *et al.*, 2015a. The two above mentioned capture antibodies were mouse monoclonal anti-NfH antibodies (Covance, USA) while the detector antibody was rabbit polyclonal anti-Neurofilament 200 (N4142; Sigma, UK). The reporter antibody was horseradish peroxidase (HRP)-labelled swine polyclonal anti-rabbit antibody (P0217; DAKO, Denmark). For the rest of the manuscript we will therefore refer to the sum of the effect of SMI34 and SMI35 as NfH. As for NfL, extensive analytical refinement was undertaken to assess analytical sensitivity, lower detection levels and linearity (Lu *et al.*, 2012). This work included the study of the reported hook effect when testing linearity of NfH expression in dilution experiment with a standard protein. Pre-analytical treatment with urea was employed to overcome the hook effect which results from a lack of parallelism due

to endogenous binding to heterogenous aggregates (protein fragments and IgG) (Lu *et al.*, 2011). The quality control sample was used to record the inter-assay variation and for additional one-point calibration as described [Petzold *et al.* 2010]. The assay protocol is summarised in the *Supplementary table 1*.

Supplementary table 1: Protocol for the NfH ELISA.

Capture antibody	Load 100 µl of Capture antibody (Covance SMI-34R or SMI-35R in carbonate buffer[1]; v/w, 2/10000) into a 96-well microtitre plate*.<br>Incubate the plate at 4°C over night.
Block the plate	Decant the Capture antibody.<br>Rinse the plate twice with 150 µl wash solution[3].<br>Block the plate with 100 µl blocking solution[4] at RT for 1 hour on the shaker.
Sample preparation	Add 5 µl of original plasma into 35 µl of $Barb_2EDTA$ buffer[2] containing 0.5 M urea.<br>Well-mixed and incubate at RT for 1 hour----diluted plasma.
Sample loading	Decant the blocking solution.<br>Rinse the plate twice with 150ul wash solution.<br>Load 95 µl of sample diluent[5] into each well of the plate.<br>Apply 5 µl of NfH standard (ranging from 0-100 ng/ml), the **diluted and preincubated plasma**, and the quality control sample in duplicates.<br>Incubate at RT for 1 hour on the shaker.
Detector Antibody	Decant the samples.<br>Wash the plate with 150 µl wash solution for 5 minutes on the shaker for 3 times.<br>Load 100 µl of detector antibody (Sigma Rabbit anti-neurofilament 200 in sample diluent; v/w, 10/10000) into the plate.<br>Incubate at RT for 1 hour on the shaker.
Reporter Antibody	Decant the detector antibody.<br>Wash the plate with 150 µl wash solution for 5 minutes on the shaker for 3 times.<br>Load 100 µl of reporter antibody (DAKO Swine anti-rabbit HRP-linked antibody in sample diluent; v/w, 10/10000) into the plate.<br>Incubate at RT for 1 hour on the shaker.
Chemiluminescence read-out	Decant the reporter antibody.<br>Wash the plate 6 times with wash solution for 5 minutes on the shaker.<br>Load 100 µl of TMB into the plate.<br>Incubate at RT for 20 minutes on the shaker in the dark.<br>Stop the reaction with 50 µl of 1M HCL.<br>Read the plate at 450 nm, with 750 nm as the reference wavelength.

*All loading volume refers to volume adding into each well of the microtire plate.

[1]Carbonate Buffer (pH 9.6): 13.85g anhydrose sodium carbonate and 26.10g sodium hydrogen carbonate in per litre distilled water

[2]$Barb_2EDTA$ buffer (pH 8.6): 13.1g sodium barbitone, 2.1g barbitone, and 0.25g EDTA in per litre distilled water

[3]Wash solution: 0.2% bovine serum albumin (BSA) and 0.05% Tween 20 in $Barb_2EDTA$ buffer

[4]Blocking solution: 2% BSA in $Barb_2EDTA$ buffer

[5]Sample diluent: 0.2% BSA in Barb2EDTA buffer

Subgroup analyses for disease progression

To assess whether clinical variables used to stratify our ALS patients may introduce a bias and affect neurofilament molar ratio calculations, we tested if any statistically significant difference was detectable in ALS subsets at the extremes of progression rate (i.e. fast progressor and slow progressors). **Supplementary table 1** shows that there is no significant difference between fast and slow progressors in terms of age at onset and sampling, smoking habits or cognitive involvement. This implies there is no potential covariate for influencing change in stoichiometry between these two sub-groups of patients.

The progression rate at time of Nf isoform sampling, baseline, was significantly related to NfM (GLM, p=0.03), NfH (GLM, p=0.03), but not NfL (GLM, p=0.13) concentrations (**Supplementary table 2**). The Bonferroni corrected p-value for the 3 progression groups calculates to 0.016. According to the Bonferroni corrections the post-hoc analyses revealed significantly higher NfM levels in fast progressors if compared to slow progressors (p=0.0074); as were NfH levels (p=0.0074).

Supplementary table 2. Clinical features and exposure: fast versus slow progressors. Continuous variables were presented with median (IQR).

Clinimetrics	fast progressors	slow progressors	p-value
**Age of onset** (years)	68.88 (59.61, 72.00)	65.89 (53.53, 68.67)	0.3786
**Age at sampling** (years)	69.60 (60.90, 73.90)	67.5 (60.53, 71.95)	0.8044
**Diagnostic latency** (months)	6.01 (2.96, 9.36)	20.6 (10.31, 37.01)	< 0.001
**Disease duration at sampling** (months)	10.51 (8.11, 14.14)	36.65 (18.83, 62.12)	< 0.001
**El-Escorial at baseline** (1: definite, 2: probable, 3: possible, 4: lab-supported probable)	2 (1, 2)	3 (1.75, 3)	0.0503
**Cognitive involvement** yes / no	7 / 20	7 / 18	0.273
**Smoking** (smokers / ex / non smokers)	3 / 7 / 17	5 / 7 / 15	0.97

Supplementary table 3. Summary of plasma concentration of NfL, NfM, and NfH in ALS patients and in controls. Plasma Nf levels of controls and ALS clinical subgroups were presented as Median (IQR).

Study subjects		NfL	NfM	NfH^{SMI34}	NfH^{SMI35}	Total NfH
**Controls (n=29)**		29.83 (19.68, 64.27)	2035.98 (1109.4, 3427.1)	15.68 (5.08, 29.67)	10.24 (6.54, 14.64)	30.43 (16.58, 42.99)
**ALS patients (n=60)**		99.93 (54.62, 202.1)	1631.53 (785.16, 3034.99)	17.17 (8.69, 25.28)	11.62 (4.18, 21.61)	28.83 (15.83, 45.81)
**Gender in ALS patients**	**female (n=27)**	125.70 (59.31, 266.07)	1802.92 (1264.15, 3461.71)	16.45 (0.74, 23.24)	12.12 (3.72, 29.48)	30.21 (12.21, 50.70)
	**male (n=33)**	79.16 (53.22, 154.22)	1563.09 (500.46, 2583.1)	18.66 (9.52, 27.24)	11.49 (4.95, 20,18)	28.13 (17.36, 45.05)
**Age at onset in ALS patients (years)**	**< 60 (n=19)**	104.50 (59.31, 172.35)	1501.34 (1064.98, 2146.97)	8.69 (1, 19.42)	11.49 (3.75, 16.13)	22.44 (8.44, 35.19)
	**60 - 69 (n=26)**	89.11 (53.54, 199.68)	1371.22 (529.67, 3074.21)	15.97 (9.52, 21.99)	10.76 (2.9, 20.18)	27.88 (16.41, 39.98)
	**70 - 79 (n=10)**	116.04 (58.46, 195.22)	2369.31 (1723.8, 3215.92)	25.52 (17.37, 36.77)	11.75 (3.71, 30.09)	42.59 (26.5, 58.46)
	**> 80 (n=5)**	220.17 (56.24, 356.829)	4322.72 (528.72, 5021.42)	31.47 (14.70, 51.58)	40.51 (16.06, 69.79)	81.24 (35.56, 111.93)
**Smoking in ALS patients**	**yes, current (n=13)**	123.13 (51.58, 204.82)	1501.34 (488.86, 2384.66)	16.97 (0.19, 19.56)	8.02 (1.22, 15.19)	27.88 (1.39, 33.71)
	**ex-smoker (n=13)**	146.36 (64.58, 265.86)	2652.19 (1149.24, 4552.01)	17.44 (8.86, 34.01)	11.49 (6.57, 21.59)	22.44 (13.92, 52.48)
	**no (n=33)**	82.33 (54.41, 185.11)	1501.34 (798.51, 2500.08)	17.17 (8.71, 31.34)	12.52 (3.97, 27.55)	32.12 (15.90, 51.14)
**Disease duration at sampling (years)**	**Within 1st year (n=18)**	140.28 (58.36, 233.84)	1283.16 (535.00, 2502.72)	18.46 (9.92, 36.77)	18.89 (6.50, 32.5)	35.99 (20.64, 62.44)
	**2nd year (n=16)**	133.23 (61.68, 216.14)	1631.53 (81.27, 3705.87)	16.97 (7.88, 25.28)	10.24 (2.33, 14.53)	26.89 (12.91, 45.61)
	**3rd year (n=11)**	95.89 (54.83, 250)	1628.46 (1332.96, 3499.3)	15.97 (1.19, 19.30)	12.52 (1.22, 18.53)	28.83 (10.33, 37.91)
	**After 3rd year (n=15)**	59.31 (48.09, 154.35)	2146.97 (1381.49, 2486.15)	15.73 (1, 19.93)	11.71 (3.75, 21.61)	28.13 (12.61, 45.81)

**Site of symptoms onset**	**bulbar (n=16)**	146.36 (53.54, 250)	1501.34 (611.45, 2652.19)	16.97 (8.69, 29.22)	14.53 (1.22, 29.39)	31.5 (15.97, 55.17)
	**limb (n=44)**	95.89 (54.83, 197.86)	1846.44 (791.84, 3293.99)	17.31 (8.45, 25.01)	11.24 (4.27, 21.33)	28.07 (15.58, 45.33)
**El Escorial diagnosis at sampling**	**definite (n=14)**	147.28 (58.69, 178.73)	1597.31 (859.39, 2451.03)	13.85 (6.76, 18.82)	11.74 (2.05, 16.73)	23.62 (8.99, 37.85)
	**probable (n=29)**	125.70 (58.95, 220.17)	2205.35 (968.26, 3498.53)	16.71 (7.21, 23.28)	8.07 (1.32, 28.47)	27.38 (13.68, 49.99)
	**possible (n=11)**	61.74 (48.09, 265.66)	1057.72 (491.2, 2285.73)	19.93 (7.88, 31.22)	15.88 (11.49, 37.53)	35.19 (19.37, 81.24)
	**lab-supported probable (n=6)**	67.82 (48.86, 152.07)	1922.64 (575.99, 3925.15)	36.72 (13.88, 43.44)	11.75 (5.91, 17.31)	45.71 (27.65, 56.5)
**ALS stage at sampling (based on ALSFRS-r score)**	**early: 40 - 48 (n=18)**	82.33 (53.46, 154.08)	1628.46 (560.59, 3074.21)	18.69 (9.52, 32.8)	12.37 (5.59, 19.63)	31.5 (14.88, 55.14)
	**intermediate: 25 - 39 (n=34)**	99.73 (59.46, 178.31)	1532.22 (762.85, 2411.41)	17.31 (7.94, 24.48)	10.54 (2.15, 24.25)	28.48 (15.75, 44.77)
	**late: < 24 (n=8)**	211.17 (52.38, 299.98)	2969.31 (1303.03, 4094.15)	14.23 (2.72, 19.53)	15.48 (5.69, 26.95)	25.74 (13.45, 46.38)
**Progression rate at baseline**	**fast: > 1 (n=17)**	156.48 (95.81, 313.51)	2652.19 (1294.76, 4024.76)	17.44 (9.1, 19.79)	8.11 (5.34, 26.58)	27.88 (16.33, 45.67)
	**intermediate: 0.5 - 1 (n=20)**	64.92 (53.54, 172.35)	1345.97 (279.99, 2188.95)	15.73 (0.92, 29.21)	11.35 (1.32, 24.46)	27.86 (6.71, 49.09)
	**slow: < 0.5 (n=23)**	79.10 (52.06, 144.15)	1784.42 (805.19, 2493.11)	17.93 (9.76, 24.92)	12.45 (9.03, 21.55)	31.54 (20.84, 48.15)
**Progression rate at the last visit**	**fast: > 1 (n=18)**	197.86 (134.19, 311.43)	2452.9 (833.93, 3886.0)	12.48 (8.35, 19.76)	11.61 (4.54, 25.71)	27.88 (16,84, 42.14)
	**intermediate: 0.5 - 1 (n=23)**	74.11 (56.24, 148.18)	1391.76 (295.41, 2588.24)	15.85 (1.97, 26.26)	7.32 (1.22, 15.86)	22.79 (11.57, 44.77)
	**slow: < 0.5 (n=19)**	68.87 (47.69, 116.96)	1630.0 (755.61, 2335.83)	19.39 (11.45, 39.78)	12.88 (9.96, 23.97)	36.78 (22.44, 64.19)

Supplementary Figure 1 The concentrations of neurofilament isoforms are shown for patients with ALS and controls for (A) NfL [g/L], (B) NfM [g/L] and (C) NfH [g/L]. Because of the non-Gaussian distribution of Nf isoform levels the non-parametric Kruskal-Wallis test was used for this post-hoc the comparison of controls and patients. The plasma NfL levels were significantly higher in samples from patients with ALS compared to controls (p<0.0001). There was no statistical significant difference for NfM (p=0.3) or NfH (p=0.8).

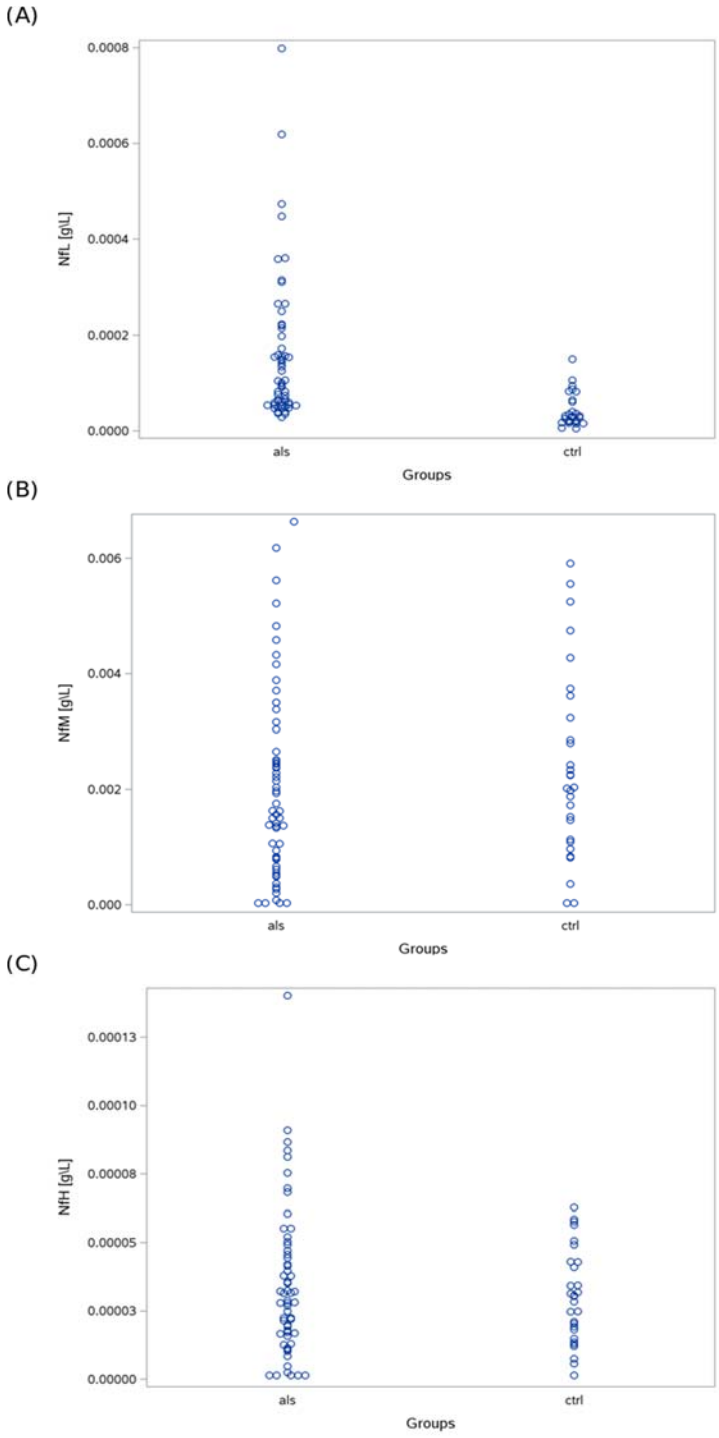

Supplement: Supplementary file 4 — Table S1. Protocol for the NfH ELISA. Table S2. Clinical features and exposure: fast versus slow progressors. Continuous variables were presented with median (IQR). Table S3. Summary of plasma concentration of NfL, NfM, and NfH in ALS patients and in controls. Plasma Nf levels of controls and ALS clinical subgroups were presented as Median (IQR). [file JNC-146-631-s004.pdf]
